# Spatial turnover of soil viral populations and genotypes overlain by cohesive responses to moisture in grasslands

**DOI:** 10.1073/pnas.2209132119

**Published:** 2022-11-02

**Authors:** Christian Santos-Medellín, Katerina Estera-Molina, Mengting Yuan, Jennifer Pett-Ridge, Mary K. Firestone, Joanne B. Emerson

**Affiliations:** ^a^Department of Plant Pathology, University of California, Davis, CA 95616;; ^b^Department of Environmental Science, Policy, and Management, University of California, Berkeley, CA 94720;; ^c^Physical and Life Sciences Directorate, Lawrence Livermore National Laboratory, Livermore, CA 94550;; ^d^Life & Environmental Sciences Department, University of California, Merced, CA 95343

**Keywords:** soil virome, soil microbiome, distance–decay relationship, relic DNA

## Abstract

Viruses shape microbial communities, food web dynamics, and carbon and nutrient cycling in diverse ecosystems. However, little is known about the patterns and drivers of viral community composition, particularly in soil, precluding a predictive understanding of viral impacts on terrestrial habitats. To investigate soil viral community assembly processes, here we analyzed 43 soil viromes from a rainfall manipulation experiment in a Mediterranean grassland in California. We identified 5,315 viral populations (viral operational taxonomic units [vOTUs] with a representative sequence ≥10 kbp) and found that viral community composition exhibited a highly significant distance–decay relationship within the 200-m^2^ field site. This pattern was recapitulated by the intrapopulation microheterogeneity trends of prevalent vOTUs (detected in ≥90% of the viromes), which tended to exhibit negative correlations between spatial distance and the genomic similarity of their predominant allelic variants. Although significant spatial structuring was also observed in the bacterial and archaeal communities, the signal was dampened relative to the viromes, suggesting differences in local assembly drivers for viruses and prokaryotes and/or differences in the temporal scales captured by viromes and total DNA. Despite the overwhelming spatial signal, evidence for environmental filtering was revealed in a protein-sharing network analysis, wherein a group of related vOTUs predicted to infect actinobacteria was shown to be significantly enriched in low-moisture samples distributed throughout the field. Overall, our results indicate a highly diverse, dynamic, active, and spatially structured soil virosphere capable of rapid responses to changing environmental conditions.

## Significance

Through infection and mortality of microbiota, viruses have myriad impacts on host metabolism, evolution, and Earth’s biogeochemical cycles. The sheer abundance of soil viruses hints at their likely importance in terrestrial ecosystems, yet the factors shaping soil viral diversity are poorly understood. Here, we show that grassland viral communities exhibited robust spatial structuring across one field, suggesting strong dispersal limitations at the local scale. Further, a shift in viral community composition accompanied a decrease in soil moisture, whereby phages predicted to infect abundant actinobacteria were enriched. Thus, despite spatial turnover, viruses responded cohesively to changing environmental conditions, suggesting the potential for a predictive understanding of soil virosphere dynamics and impacts on terrestrial processes.

With an estimated area of 52.5 million km^2^ (1), grasslands are major contributors to the cycling (2) and storage (3) of soil organic carbon. Soil microorganisms play key roles in these biogeochemical processes (4, 5), and, by infecting soil microbiota (6, 7), viruses likely have substantial impacts on carbon dynamics (8). The potential importance of viruses in soils (9–12), together with their measured high abundance—10^7^ to 10^10^ virus-like particles per gram of soil (9)—and improvements in our ability to sequence and track soil viral genomes (8, 13) have led to a renewed flurry of investigations into soil viral ecology (14–21). However, despite a new appreciation for the vast diversity of soil viruses (14–18, 22), little is known about the factors that govern soil viral community assembly, precluding a robust and predictive understanding of viral impacts on terrestrial ecosystem processes.

Soils are physically, chemically, and biologically heterogeneous (23). The intricate network of aggregates and pore spaces that constitutes the soil matrix (24) not only sustains a varying landscape of edaphic properties but also restricts the movement of microorganisms (25). Such environmental gradients and dispersal limitations often lead to the spatial structuring of microbial diversity (26). For example, distance–decay of community similarity, a biogeographical relationship in which communities become more compositionally dissimilar as the spatial distance between them increases (27), has been reported for soil bacteria across multiple scales and habitats (28–30). Yet, despite evidence of local adaptation of soil bacteriophages to host strains at the centimeter scale (31), the extent to which the soil virosphere is also spatially structured has not been thoroughly explored. An understanding of the compositional turnover of viral communities across space is, therefore, necessary to begin unraveling the spatial constraints of host–virus interactions in soil.

Given its multifaceted role as a resource, solvent, and transport medium, water is a central regulator of the activity, abundance, and dispersal of soil microorganisms (32). In Mediterranean climate grasslands, where wet winters follow dry summers, seasonal rainfalls dictate the compositional dynamics of soil microbiomes (33). Thus, the forecasted alteration of precipitation patterns due to climate change (34) could impact soil trophic networks and their contributions to the biogeochemical processes in these habitats (35). Rainfall manipulation experiments have shown that reduced precipitation can reshape soil bacterial community composition (36, 37). Whether these shifts are coupled to changes in the soil virosphere remains unknown, although recent observations suggest that water availability could be a major driver of soil viral community assembly. For example, a comparison of three distinct grassland sites revealed a significant correlation between soil moisture and viral richness (21), and a laboratory study identified a substantial shift in viral diversity triggered by the wetting of dry biocrust soil (20). As such, characterizing the compositional response of soil viral communities to reduced precipitation can help us understand the potential impact of a changing environment on host–virus interactions.

In this study, we generated viral-size-fraction metagenomes (viromes) to profile the double-stranded DNA (dsDNA) viruses inhabiting a Mediterranean grassland exposed to rainfall-exclusion treatments. The comprehensive access to soil viral diversity enabled by this viromics approach (14, 38) allowed us to characterize the spatial turnover of viral populations and genotypes at a local scale and dissect viral community responses to changes in soil moisture. Complementary analyses of 16S ribosomal RNA (rRNA) gene amplicon sequencing data from the same samples allowed for comparisons of community assembly patterns for viruses and their bacterial and archaeal hosts. Our results revealed a spatially structured soil virosphere that can respond cohesively to reduced precipitation.

## Results and Discussion

**Fig. 1.**
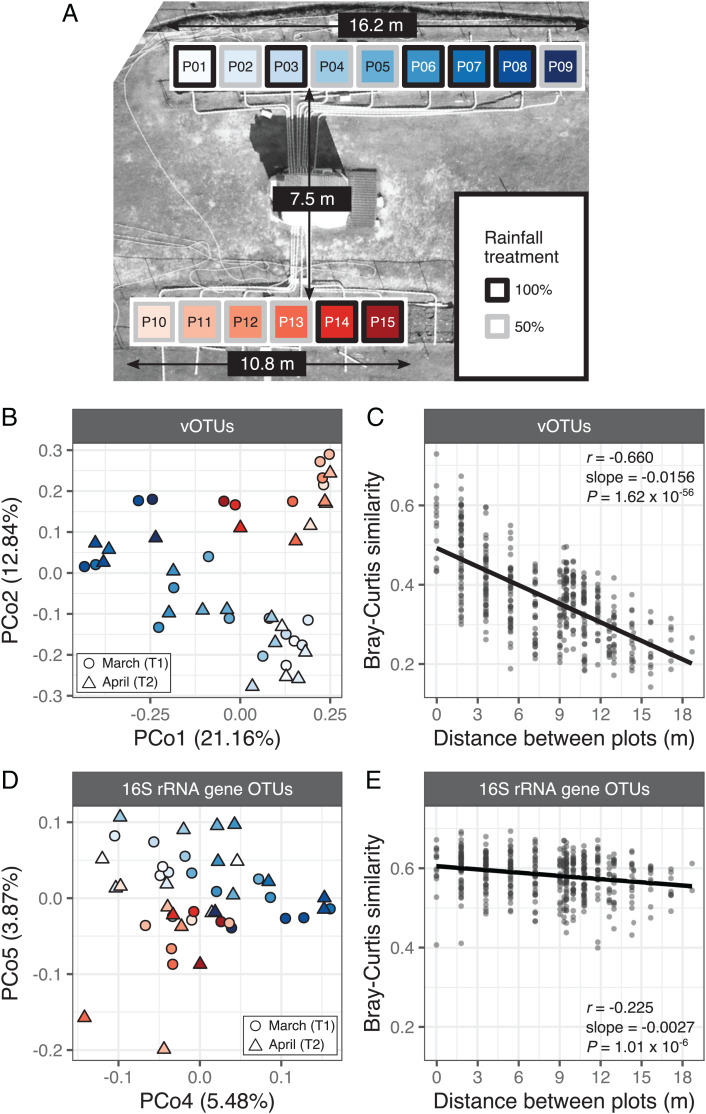
Spatial structuring of viral and prokaryotic communities in a Mediterranean grassland. (*A*) Aerial view of the field site. Colored squares mark the locations of the sampled plots within the upper (blue) and lower (red) blocks. Square outlines indicate the rainfall manipulation regime assigned to each plot. Differences in font color are for legibility only. (*B* and *D*) Unconstrained analysis of principal coordinates performed on (*B*) vOTU and (*D*) 16S rRNA gene OTU Bray–Curtis dissimilarities. *B* displays the first and second axes and *D* displays the fourth and fifth axes, as they best captured the spatial structuring in (*B*) viral and (*D*) bacterial and archaeal communities. Color reflects the plot from which the sample was collected and corresponds to the gradient palette in *A*. Point shape represents the collection time point. Axis labels indicate the percentage of total variance explained. (*C* and *E*) Relationship between Bray–Curtis similarity and spatial distance in (*C*) viral communities and (*E*) bacterial and archaeal communities. Each point represents a pair of samples, and the spatial distance between them was measured as the length of the line connecting the centers of the corresponding plots. Pairs of samples involving different time points were excluded from these analyses. Trend lines display the least squares linear regression model. Inset statistics correspond to the Pearson’s correlation coefficient (*r*), the linear regression slope, and the associated *P* value.

To characterize dsDNA viral diversity and investigate viral community compositional patterns in Mediterranean grasslands, we collected surface (0 to 15 cm) soil samples from a field site at the Hopland Research and Extension Center in northern California (Fig. 1*A*). Soils were harvested from 22 subplots distributed across 15 experimental plots arranged in two separate blocks (*SI Appendix*, Fig. 1 *A* and *B*). These plots have been maintained since 2017 with either 100% or 50% of the average historical precipitation via rainfall-excluding shelters and controlled irrigation (39) (*SI Appendix*, Fig. 1 *C* and *D*). Samples were collected from densely rooted locations (*SI Appendix*, Fig. 1*B*) at two time points (March and April, T1 and T2, respectively) during the 2020 growing season of *Avena barbata* (slender wild oat), the naturalized annual grass that dominates the site (*SI Appendix*, Fig. 1*E*).

Soil viral community composition was profiled via 44 viral-size-fraction metagenomes. These viromes were generated from frozen soil samples, and their processing did not include the DNase digestion typically performed to remove extracellular DNA contaminants prior to virion DNA extraction (see *Materials and Methods*). Yet, despite potential overrepresentation of microbial sequences, 18,040 out of 30,238 contigs assembled across viromes were identified as viral by VIBRANT (40), a viral enrichment comparable to previous reports from non-DNase-treated viromes generated from fresh soil samples (38). Viral contig clustering at ≥95% average nucleotide identity (ANI) across 85% of the alignment fraction yielded 6,088 approximately species-level viral operational taxonomic units (vOTUs) that served as references for read recruitment to establish vOTU relative abundances (41). After removing vOTUs exclusively detected in single viromes and excluding one virome due to poor vOTU recovery, the final dataset consisted of 43 viromes and 5,315 vOTUs (Dataset S1).

### Viral Community Composition Followed a Stronger Distance–Decay Relationship than Bacterial and Archaeal Communities at the Field Scale.

**Fig. 2.**
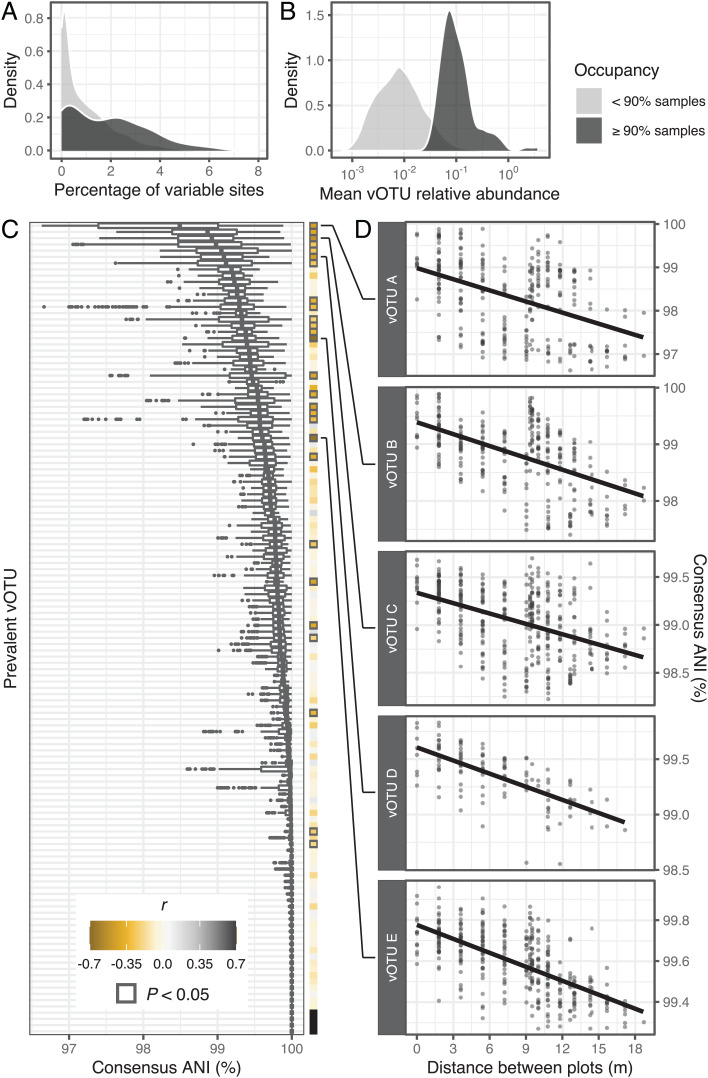
Spatial structuring of viral population microdiversity. (*A* and *B*) Kernel density plots showing the distributions of (*A*) microdiversity (measured as the percentage of polymorphic sites in a vOTU sequence) and (*B*) mean relative abundance within prevalent (≥90% occupancy) and nonprevalent (<90% occupancy) vOTUs. (*C*) Distributions of ANIs for each prevalent vOTU, calculated between pairs of sample-specific vOTU consensus sequences. Each box plot corresponds to a single vOTU, and the *y* axis is in rank order (ascending from top to bottom) of the median ANI value for each vOTU. Boxes display the median and interquartile range (IQR), and data points farther than 1.5× IQR from box hinges are plotted as outliers. The heat map on the right shows the Pearson's correlation coefficients between consensus ANI and spatial distance. Bold outlines indicate a significant *P* value (<0.05) for the correlation after multiple comparisons correction (Holm algorithm). Filled black squares correspond to vOTUs with no variation across samples (i.e., all ANIs were equal to 1). (*D*) The top five vOTUs with the most significant correlations (lowest *P* values) between consensus ANI and spatial distance. Each point represents a pair of samples, and the spatial distance between them was measured as the length of the line connecting the centers of the corresponding plots. Pairs of samples involving different time points were excluded from these analyses. The trend line displays the least squares linear regression model. Note that vOTUs are defined in part by sharing ≥95% ANI (see *Materials and Methods*), so within-vOTU ANI values will necessarily be ≥95% ANI. Also note subtle differences in the *y* axis range across graphs.

**Fig. 3.**
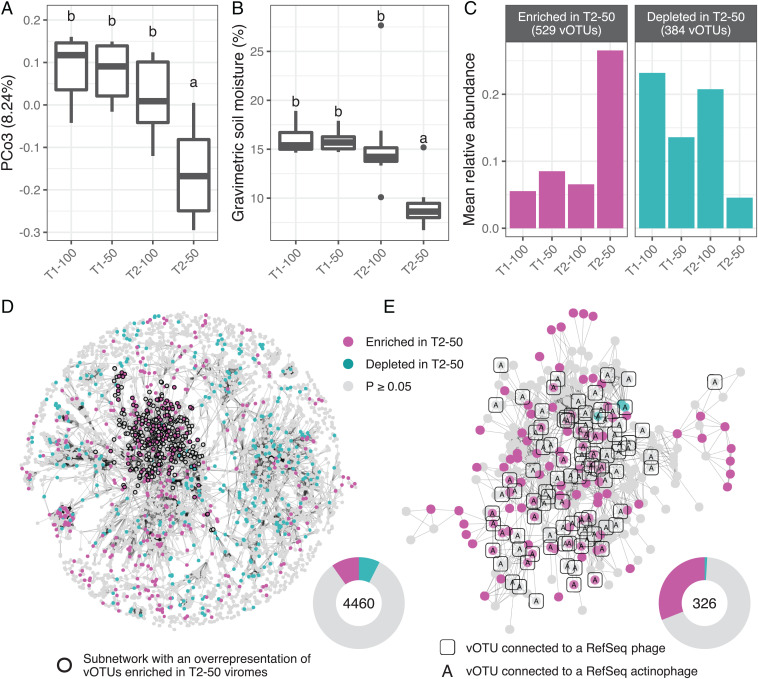
Viral community trends associated with soil moisture content. In *A–C*, samples are grouped along the *x* axis by collection time point (T1 and T2) and precipitation regime (100% and 50%). (*A*) Distribution of scores along the third axis of a PCoA performed on vOTU Bray–Curtis dissimilarities. The *y* axis label indicates the percentage of total variance explained. The first two axes of the same analysis are shown in Fig. 1*B*. (*B*) Gravimetric soil moisture contents. Boxes display the median and interquartile range (IQR), and data points farther than 1.5× IQR from box hinges are plotted as outliers. In *A* and *B*, different letters indicate significantly different sample groupings (*P* < 0.05), as determined by two-tailed Tukey’s range tests. (*C*) Summed mean relative abundances of the sets of vOTUs detected as indicator species differentiating T2-50 communities from the rest of the viromes. Facets distinguish indicator vOTUs that were relatively enriched or depleted, respectively, in T2-50 viromes. (*D*) Gene-sharing network displaying significant overlaps in predicted protein content (edges) between vOTUs (nodes). Node color shows whether a vOTU was an indicator species enriched or depleted in T2-50 samples or not an indicator species (defined by *P* values below or above 0.05, respectively, from an indicator value permutation test). Bold outlines highlight a subnetwork of all local neighborhoods with a significant overrepresentation of vOTUs enriched in T2-50 viromes (*SI Appendix*, Fig. S6 *B* and *C*). (*E*) Zoomed-in version of the subnetwork highlighted in *D*. Nodes surrounded by squares correspond to vOTUs with a significant overlap in their predicted protein contents with any of 971 RefSeq phage genomes, according to the network analysis shown in *SI Appendix*, Fig. S8. All such RefSeq phage genomes with significant links to this subnetwork were from phages isolated on Actinobacteria hosts, indicated by tagging vOTU nodes linked to RefSeq actinophages with the letter “A.” In *D* and *E*, inset donut plots on the lower right show the total number of vOTUs in the displayed network (center), along with the proportions of the indicator and nonindicator vOTUs in that network (fractions of the circle). Network visualization layouts were generated with the Fruchterman–Reingold algorithm.

Viral community beta-diversity patterns were largely explained by the spatial arrangement of the field plots, as evidenced by a longitudinal gradient captured by the first axis of a principal coordinates analysis (PCoA) and the separation of upper and lower field blocks along the second PCoA axis (Fig. 1*B*). In contrast, no meaningful clustering based on time point or watering treatment was observed along these two axes (*SI Appendix*, Fig. 2 *A* and *B*). A permutational multivariate analysis of variance (PERMANOVA) further confirmed the predominant role of spatial structuring on viral community composition relative to other experimental factors (*SI Appendix*, Table 1). Additionally, we identified a significant negative correlation between viral Bray–Curtis similarity and spatial distance between plots (Fig. 1*C*), indicating that distance–decay relationships were a key driver of viral community composition. These trends were driven, in part, by substantial differences in vOTU detection patterns across viromes: of 5,135 vOTUs, 50% were detected in 9 or fewer of the 43 viromes (*SI Appendix*, Fig. 3*A*). Moreover, the percentage of vOTUs shared between pairs of viromes declined steeply as spatial separation increased (*SI Appendix*, Fig. 3*B*). This strong spatial structuring of viral diversity within one field is consistent with prior work in agricultural soils (14) and over larger spatial distances (42), suggesting that distance–decay relationships could be a conserved feature of the soil virosphere. For example, a recent viromic survey of five different natural reserves, including grasslands and other habitats, in northern California found that more than 90% of the dsDNA vOTUs were exclusively found in individual locations (43). Similarly, minimal overlap in soil RNA viral community composition was observed across five sites, including four grasslands, within a 3-km transect (42). Thus, the observed turnover of viral populations at the local scale in this study (Fig. 1*B*) may translate to stark compositional distinctions at the regional scale. Future studies with a broader spatial and temporal range will be needed to fully assess the prevalence of these biogeographical patterns.

To assess whether bacterial and archaeal communities displayed similar spatial patterns at our field site, we performed 16S rRNA gene amplicon profiling on total DNA extracted from the same soil samples used to generate the viromes. In contrast to the strong spatial patterns in the viral communities, collection time point was the main factor shaping prokaryotic beta-diversity, as indicated by a significant PERMANOVA (*SI Appendix*, Table 2) and a clear distinction between March and April samples along the first axis of a PCoA (*SI Appendix*, Fig. 2 *C* and *D*). While spatial structuring was also detected in the bacterial and archaeal communities (*SI Appendix*, Table 2), its effect was only evident along the fourth and fifth PCoA axes (Fig. 2*D*). Further, even though spatial distance was significantly negatively correlated with microbial community Bray–Curtis similarity (Fig. 2*E*), this association was not as pronounced as for the viral communities. Specifically, the spatial turnover rate of community similarity (the slope of the distance–decay relationship) was 5.8 times higher for viruses than for bacteria and archaea. Similar observations of spatial succession of viral but not prokaryotic communities along an 18-m gradient in an agricultural field (14) raise the possibility that stronger spatial structuring in viral relative to bacterial and archaeal communities (Fig. 1 *B* and *D*) might be a generalizable pattern across soils.

**Fig. 4.**
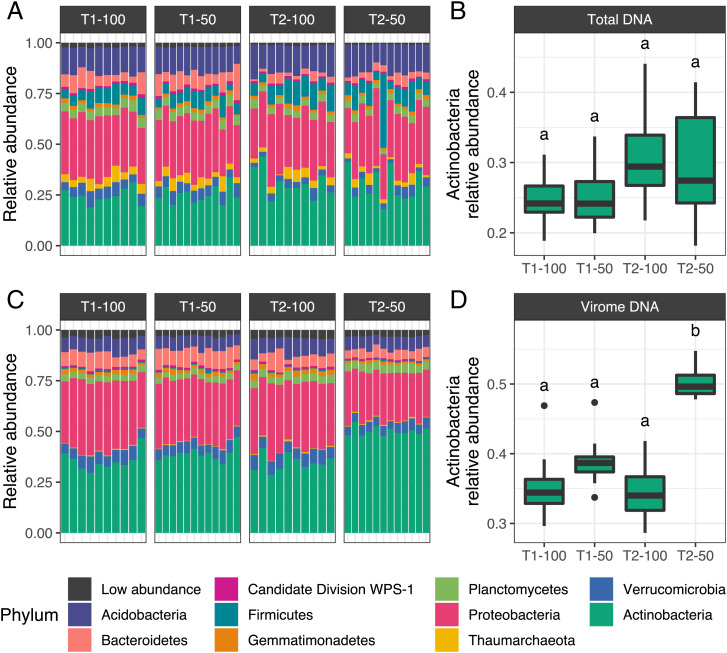
Abundance patterns of actinobacteria in total and relic DNA profiles. (*A* and *C*) Phylum abundances in 16S rRNA gene profiles from (*A*) total DNA 16S rRNA gene amplicon libraries and (*C*) virome DNA libraries. Each stacked bar plot corresponds to a sample, and the 10 most abundant phyla are colored. All other phyla are grouped in the “Low abundance” category. (*B* and *D*) Relative abundances of actinobacteria in (*B*) total DNA 16S rRNA gene amplicon libraries and (*D*) virome DNA libraries. Samples are organized by collection time point (T1 and T2) and precipitation treatment regime (100% and 50%). Boxes display the median and interquartile range (IQR), and data points farther than 1.5× IQR from box hinges are plotted as outliers. Letters above boxes indicate significantly different groupings (*P* < 0.05), as determined by pairwise Wilcoxon’s rank-sum tests. For *C* and *D*, abundances were normalized to the number of reads classified as 16S rRNA genes in each virome profile; a complementary analysis with abundances normalized to the total number of reads in each virome profile is provided in *SI Appendix*, Fig. S10.

The observed differences in distance–decay patterns between viral and prokaryotic communities suggest that the underlying assembly processes governing spatial structuring at local scales could be differentially impacting these two components of the soil microbiome. For example, viruses and cellular microorganisms likely experience distinct dispersal limitations linked to size, adsorption specificities, and transport mechanisms (44, 45). Some of the observed spatial gradients in the abiotic environment in our study (*SI Appendix*, Fig. 4*A*), such as spatially structured soil calcium concentrations (*SI Appendix*, Fig. 4*B*), are consistent with this scenario, as attachment to soil surfaces in the presence of Ca^2+^ has been shown to be significantly higher for viral particles than for bacteria (45), potentially impacting their relative movement in soil. Environmental filtering, whereby abiotic and/or biotic factors influence the distribution of microbial populations through selective pressure (26), may also be particularly relevant for viral community assembly. For instance, edaphic properties can directly affect viral viability and transport (11, 46), while viral reliance on hosts for replication renders the hosts themselves to be unavoidable environmental filters (47). Interestingly, viral beta-diversity was significantly correlated with both the abiotic environment and prokaryotic community composition (*SI Appendix*, Fig. 5 *A* and *B*), suggesting that even the dampened spatial structuring of edaphic properties (*SI Appendix*, Fig. 4*A*) and prokayrotic beta-diversity (Fig. 1*E*) could have contributed to the amplified spatial structuring of viruses in this study (Fig. 1*C*). Consistent with biotic environmental selection, RNA viral communities in grasslands differed significantly in the presence of plant litter and across soil compartments (16), and dsDNA viral communities differed along a permafrost thaw gradient (18), with patterns similar to those of their host communities in both cases (16, 48). Differences in the strength of the spatial patterns between viruses and prokaryotes could also be related to differences in the integrated temporal scales captured by DNA pools in viromes compared to total DNA (49). For example, fast viral particle decay rates and the large burst sizes (11, 50, 51) characteristic of viral replication could amplify the signal of recent viral infections in the viromes, while relic DNA (52) and DNA from dormant biota (53) could mask the signal from active microbes in total DNA. Together, our results suggest that dispersal, abiotic, and biotic factors could all contribute to soil viral community assembly patterns and, potentially, to differences between soil host and viral biogeography.

### Genomic Microdiversity of Viral Populations Tended to Be Spatially Structured.

In addition to environmental filtering and dispersal, diversification (i.e., the generation of novel genetic variation) can contribute to diversity patterns in microbial communities (26, 54, 55). To explore the role of spatial structuring on viral genotypic heterogeneity across our field site, we profiled within-population genomic variation. Briefly, using inStrain (56), we scanned all mapped reads assigned to individual vOTUs and identified polymorphic sites. Then, to assess intersample vOTU genomic similarities, we reconstructed sample-specific consensus vOTU sequences and performed pairwise ANI comparisons. Given that most vOTUs were detected in a limited number of viromes (*SI Appendix*, Fig. 3*A*), we restricted this analysis to a subset of 130 vOTUs that were detected in at least 90% of the viromes. This set of prevalent vOTUs had high levels of intrapopulation heterogeneity (Fig. 2*A*) and also consisted of some of the most abundant viral community members (Fig. 2*B*). The ANI distributions revealed a wide range of genomic variation among dominant allelic variants: While some prevalent vOTUs had mean pairwise variant similarities close to 0.95 ANI (the threshold used to define a viral population), others appeared nearly clonal across samples (Fig. 2*C*). Moreover, the microdiversity of many vOTUs was spatially structured within our field site. For 21% of the prevalent vOTUs and 54% of the 26 vOTUs that displayed the most variation (prevalent vOTUs with median ANIs <99.5%), genomic similarity displayed a significant negative correlation with spatial distance, indicating that the predominant allelic variants tended to diverge with increasing distance (Fig. 2*D* and *SI Appendix*, Table 3). Together, these results show that viral community composition and the genetic makeup of viral populations exhibited significant distance–decay relationships across our field site.

### Low-Moisture Soils Had Significantly Different Viral Communities and Were Enriched in Putative Actinophages.

Although spatial structuring emerged as the predominant driver of soil viral diversity patterns (Fig. 1 *B* and *C*), viral community composition was also shaped by the experimental precipitation treatments. In particular, the third axis of our PCoA, which accounted for 8.24% of variance in the dataset, captured a significant distinction between the April viromes from 50% precipitation plots (“T2-50” samples) and the rest of the viromes (Fig. 3*A*). Gravimetric soil moisture contents were also significantly lower for these T2-50 samples (Fig. 3*B*), a distinction that likely reflects the differential precipitation exclusion patterns preceding each collection time point. In particular, both 50% and 100% plots were fully exposed to rainfall during the month leading up to the first sample collection; in contrast, 50% treatment plots underwent a 24-d-long precipitation exclusion immediately before the second sample collection (*SI Appendix*, Fig. 1 *C* and *D*). These differences in recent precipitation likely explain why T1-50 samples had similar soil moisture to the 100% precipitation treatment samples from both time points, whereas T2-50 samples had lower soil moisture and correspondingly distinct viral communities. Together, these trends suggest that viral communities were directly or indirectly structured by changes in soil moisture during the growing season.

We next identified vOTUs with significant responses to these soil moisture patterns to assess potential commonalities among them. An indicator species analysis revealed 529 vOTUs that were significantly enriched in T2-50 viromes relative to the rest of the viromes and 384 vOTUs that were significantly depleted (Fig. 3*C*). As functional traits can be phylogenetically conserved in bacteria (57), we assessed whether analyses at higher levels of viral genome conservation might reveal a similarly cohesive response to soil moisture. Using vConTACT2 (58), we constructed a network of vOTUs (nodes), in which each edge indicated a significant overlap in predicted protein contents between a pair of vOTUs. We then adapted an algorithm designed to assess local overrepresentation of traits in biological networks (59) to characterize the network distribution of soil-moisture-responding vOTUs. Briefly, for each vOTU, we identified a local neighborhood of all vOTUs that could be reached, either directly or indirectly, via an edge path with a length shorter than the first percentile of all pairwise node distances in the network. After discarding all local neighborhoods with fewer than 10 vOTUs, we recovered 2,865 subnetworks of highly interconnected nodes with a median size of 39 vOTUs, allowing us to consider many more sizeable groups of related vOTUs than a standard vConTACT2 analysis of “genus-level” viral clusters (VCs) (15, 60), as there were only 24 VCs with at least 10 vOTUs in this dataset. Next, we performed hypergeometric tests to assess the overrepresentation of indicator vOTUs enriched or depleted in T2-50 samples within each network neighborhood. A total of 108 neighborhoods showed a significant overabundance of vOTUs consistently enriched in T2-50 samples, with 26 to 67% of vOTUs in these neighborhoods displaying this trait, compared to only 10% of vOTUs in the whole network (*SI Appendix*, Fig. 6 *A–C*). This pattern contrasted with the lack of substantial network aggregation of vOTUs depleted in T2-50 samples, as only four small, local neighborhoods displayed a significant, albeit weak, overrepresentation of this trait (*SI Appendix*, Fig. 6 *A* and *B*). Interestingly, all of the significantly T2-50 enriched trait neighborhoods were constrained to a single region in the protein-sharing network, indicating that a relatively cohesive group of related vOTUs tended to be enriched in T2-50 samples (Fig. 3*D*). Further, the indicator vOTUs within this subnetwork covered a range of detection patterns across viromes (occupancies) and were spatially distributed across the field site (*SI Appendix*, Fig. 7 *A* and *B*). This suggests that, despite the strong spatial structuring of viral communities overall, this group of genomically related vOTUs responded cohesively to changes in soil moisture, regardless of their field plot locations.

To further explore the subnetwork with a significant overrepresentation of low-moisture (T2-50)-enriched vOTUs, we performed a second protein-sharing network analysis, with all prokaryotic viral genomes in the NCBI RefSeq database. We identified edge connections between vOTUs in the low-moisture trait subnetwork and RefSeq viral genomes to assess network neighborhood trends in viral and host taxonomy (*SI Appendix*, Fig. 8 *A–C*). Of 326 vOTUs in the subnetwork, 96 were connected to at least one RefSeq viral genome, all of which were classified as Siphoviridae or as undefined viruses from the order Caudovirales (*SI Appendix*, Fig. 8*B*), both taxonomic classifications currently under consideration to be replaced by monophyletic genome-based families (61). More interestingly, all 191 RefSeq viral genomes connected to a trait subnetwork vOTU were isolated from Actinobacteria hosts, suggesting that the low-moisture-responsive vOTU subnetwork was largely composed of actinobacteriophages (Fig. 3*E*). In contrast, only 38% of all 971 vOTUs associated with RefSeq genomes across the entire network were exclusively linked to an actinobacteriophage (*SI Appendix*, Fig. 8*C*), indicating a substantial concentration of putative actinobacteriophages in the subnetwork. These results suggest that low soil moisture could have increased the activity of actinobacteria, in turn driving increased predation by actinophages.

### The Relative Enrichment of Putative Actinophages in Low-Moisture Soils Coincided with an Increase in Relic DNA from Actinobacteria.

Many actinobacteria are drought-resistant members of soil microbiomes that can increase their activity and abundance under low-moisture conditions across multiple environments (36, 62–64), including Mediterranean grasslands (33). While actinobacteria were among the most abundant members in the 16S rRNA gene amplicon profiles, there were no significant differences in their relative abundances across watering treatments or time points (Fig. 4 *A* and *B*). Further, even though the first axis of a PCoA captured a microbial community compositional shift from March to April (*SI Appendix*, Fig. 2*D*), there was no clear distinction between T2-50 prokaryotic communities and the rest of the samples (*SI Appendix*, Fig. 9). Given that microbial community sensitivity to an environmental disturbance is linked to the intensity and duration of the stressor (65), the absence of a significant effect of watering treatment on prokaryotic community composition could stem from the temporal scale of the dry-down captured in this study. In particular, the 24-d-long rainfall exclusion that 50% treatment plots experienced between the two collection time points (*SI Appendix*, Fig. 1*C*) might not have been long enough to detect the compositional shifts previously observed during more prolonged desiccation periods (33). Additionally, given that rainfall-exclusion treatments in our site started in 2017, treatment legacy effects could have also dampened the microbiome response under low-moisture conditions, as preexposure to drought can increase the resistance of soil bacteria to future desiccation events (36).

While the effects of low moisture observed in the virosphere were not recapitulated by bacterial and archaeal communities overall or by actinobacterial relative abundances specifically, it is possible that the presence of genetic material from dead and dormant cells in the total DNA profiles could have concealed underlying ecological dynamics driven by physiologically active microorganisms (52, 53). Because of its high abundance in soils (66), extracellular DNA from dead cells can introduce substantial biases in estimates of microbial abundance, especially when the turnover rate of this relic DNA is disrupted by environmental perturbations, such as bacteriophage blooms (49). To consider the relic DNA pool in our samples more directly, we recovered reads classified as 16S rRNA gene fragments from virome profiles. Given that viral enrichment in viromes was achieved via 0.22-µm filtration prior to DNA extraction, any bacterial and archaeal sequences present in these libraries likely originated from relic DNA or small (<0.22 µm) microbial cells (38, 67). Interestingly, the relative abundance of Actinobacteria 16S rRNA gene reads recovered from T2-50 viromes was significantly higher than in any other group of samples (Fig. 4 *C* and *D* and *SI Appendix*, Fig. 10). This increase in (presumably) free actinobacteria DNA, coupled with the enrichment of putative actinophages in the T2-50 subnetworks (Fig. 3*E*), suggests that actinobacteria hosts may have experienced higher infection and lysis under lower-moisture conditions, a scenario compatible with the capacity of environmental relic DNA to retain signatures of recent viral infections (68). Interestingly, actinobacteria can display enhanced transcriptional activity under drought conditions (33, 62), suggesting that the potential increase in actinobacteria–actinophage interactions observed in this study could stem from a heightened metabolic state in actinobacteria under low-moisture conditions. Future studies dissecting the functional changes that soil microbiomes undergo during reduced precipitation could help us understand the mechanisms enabling these dynamics.

## Conclusions

Here, we show that grasslands harbor an active and highly dynamic soil virosphere that is structured over space and can respond to a changing environment. The high degree of spatial turnover that we observed—within one field site during one growing season—suggests dispersal limitations for viral populations and genotypes on scales of meters and months, hinting at the potential spatial patterning of host–virus interactions in soil. Moreover, the disparity in distance–decay relationships between viral and prokaryotic communities could reflect potential differences in the assembly processes shaping these two components of the soil microbiome. The compositional shift triggered by reduced precipitation further indicates that, despite the underlying spatial structuring, groups of genomically related viruses can respond cohesively to environmental conditions, such as decreases in soil moisture, presumably by way of their hosts. Finally, the coupled enrichment of putative actinophages and relic DNA from actinobacteria under low-moisture conditions raises the possibility that reduced precipitation increased the infection and lysis of a prevalent, drought-responsive group of soil microorganisms. In summary, soil viral community assembly seems to be tightly coupled to the heterogeneous and dynamic biotic and abiotic landscape of the local environment, and it will be interesting to see how these patterns scale over more extensive temporal and spatial distances.

## Materials and Methods

### Field Experiment and Sample Collection.

Samples were collected as part of a rainfall manipulation field experiment (39) at the University of California Hopland Research and Extension Center (39° 00′ 14.6″ N, 123° 05′ 09.1″ W). The field site contained 16 plots (1.8 × 1.8 m) arranged in two separate blocks 7.5 m apart: a 16.2-m-long upper block with nine plots and a 12.6-m-long lower block with seven plots (*SI Appendix*, Fig. 1*A*). Plot boundaries were delimited by 1-m-deep vertical plastic liners, installed in the spring of 2017, that limited water transfer between adjacent soils. Each plot contained eight circular subplots (40-cm diameter) delineated by 15-cm-deep polyvinyl chloride collars (*SI Appendix*, Fig. 1*B*). Starting in 2017 and continuing until 2020, plots were exposed to two precipitation regimes, where the amount of water received by each plot was adjusted to match 100% or 50% of the average historical precipitation at the site. Differential watering was achieved by the periodic deployment of rainfall-excluding shelters (*SI Appendix*, Fig. 1*C*) and by controlled irrigation of individual plots (*SI Appendix*, Fig. 1*D*). For this study, soils were harvested from 22 subplots distributed across 15 of the 16 plots (*SI Appendix*, Fig. 1*A*). All of these subplots were segmented in two halves by a 15-cm-deep Plexiglas divider (*SI Appendix*, Fig. 1*B*), and they were located within a 60-cm radius from the center of each plot. Sample collections were performed on 13 March and 14 April 2020 (T1 and T2, respectively) during the active growth phase of *A. barbata* (*SI Appendix*, Fig. 1*E*). At each time point, half of each subplot was destructively harvested. Samples were processed by removing any visible roots, homogenizing the soil, and storing the soil at −80 °C until further processing. For soil moisture measurements, separate fresh soil subsamples were collected and processed immediately.

### Virome DNA Extraction, Library Construction, and Shotgun Sequencing.

Due to the COVID-19 2020 lockdown, we could not perform virome extractions on fresh samples as intended and instead stored soils at −80 °C until processing. Soil virions were enriched through filtration and concentration prior to DNA extraction, following a modified version of a previously published protocol (69). For each sample, 10 g of soil were resuspended in 10 mL of protein-supplemented phosphate-buffered saline solution (PPBS: 2% bovine serum albumin, 10% phosphate-buffered saline, 1% potassium citrate, and 150 mM MgSO_4_). To elute virions, soil suspensions were vortexed until homogenized, placed on an orbital shaker (10 min, 400 rpm, 4 °C), and centrifuged (10 min, 3,095 × *g*, 4 °C). Supernatants were recovered and stored briefly at 4 °C, while pellets were resuspended in 10 mL of fresh PPBS for back-extraction of the remaining soil. This process was repeated for a total of three rounds of extraction of the same soil. Supernatants from the same sample were then pooled and centrifuged three times (10 min, 10,000 × *g*, 4 °C), retaining the supernatant and discarding the pellet each time to remove residual soil particles. Purified supernatants were then filtered through a 0.22-µm polyethersulfone membrane to remove cells. Eluted virions in the filtrate were concentrated via ultracentrifugation (2 h 25 min, 32,000 × *g*, 4 °C) in an Optima LE-80K ultracentrifuge with a 50.2 Ti rotor (Beckman-Coulter). Supernatants were removed, and pellets were resuspended in 100 µL of ultrapure water. As previously shown (38, 70), the DNase treatment step that serves to remove free DNA at this stage is not compatible with samples stored frozen (we suspect that this is because freezing compromises virions), so we were unable to perform a DNase treatment. We have previously shown that non-DNase-treated soil viromes still successfully enrich the viral signal relative to total metagenomes and capture the same ecological trends as DNase-treated viromes from the same samples (38).

DNA was extracted from the viral fraction with the DNeasy PowerSoil Pro kit (Qiagen), following the manufacturer’s protocol, with the addition of a 10-min incubation at 65 °C prior to the bead-beating step. Shotgun metagenomic libraries were constructed with the DNA Hyper Prep kit (Kapa Biosystems-Roche), and paired-end sequencing (150 bp) was performed on the NovaSeq S4 platform (Illumina).

### Total DNA Extraction, Amplicon Library Construction, and Sequencing.

Total DNA was extracted from 0.25 g of soil with the DNeasy PowerSoil Pro kit (Qiagen), following the manufacturer’s instructions, with the addition of a 10-min incubation at 65 °C prior to the bead-beating step. Construction of amplicon libraries followed a previously described dual-indexing strategy (71, 72). Briefly, universal primers 515F and 806R were used to target the V4 region of the 16S rRNA gene. Amplifications were performed with the Platinum Hot Start PCR Master Mix (Thermo Fisher) following the Earth Microbiome Project’s PCR protocol (73): an initial denaturation step at 94 °C for 3 min, 35 cycles of 94 °C for 45 s, 50 °C for 60 s, and 72 °C for 90 s, and a final extension step at 72 °C for 10 min. To account for any potential amplification of reagent contaminants (74), we used a DNA-free control (molecular-grade water processed through the same DNA extraction protocol) as a template to generate a blank library. Libraries were cleaned with AmpureXP magnetic beads (Beckman Coulter), quantified (Qubit 4 fluorometer; Thermo Fisher), and pooled in equimolar concentrations. Paired-end sequencing (250 bp) was performed on the MiSeq platform (Illumina).

### Soil Chemistry and Moisture Measurements.

Soil moisture was calculated as the ratio of mass of water per mass of dry soil. While soil moisture was originally measured for all samples, data for a subset of 11 March samples (five from 100% plots and six from 50% plots) were lost and could not be included in downstream analyses. Soil chemistry profiling was performed by Ward Laboratories: Soil pH and soluble salts were measured using a 1:1 soil:water suspension; soil organic matter was measured as the percentage weight loss on ignition; nitrate was measured via a KCl extraction; potassium, calcium, magnesium, and sodium were measured via an ammonium acetate extraction; zinc, iron, manganese, and copper were measured via a DTPA extraction; phosphorus was measured via the Olsen method; and sulfate was measured via a Mehlich-3 extraction. Soil chemistry measurements were only performed on the set of 22 soil samples collected in March (T1).

### Bioinformatic Processing.

#### Virome processing.

We used Trimmomatic v0.33 (75) to remove Illumina adapter sequences and quality-trim reads (minimum q-score of 30 evaluated on 4-base sliding windows; minimum read length of 50) and BBDuk v38.82 (76) to remove PhiX sequences. Next, we generated de novo assemblies of individual libraries with MEGAHIT v1.2.9 (77) in metalarge mode (–k-min 27 –k-max 127 –k-step 10), using a contig minimum size threshold of 10,000 bp. Assembled contigs were then classified as viral with VIBRANT v1.2.1 (40) in virome mode. Consistent with established best practices (41), the resulting viral contigs were dereplicated into nonredundant vOTUs with dRep v3.2.2 (78), using the following parameters: a threshold of ≥95% ANI across ≥85% alignment fraction (−sa = 0.95, −nc = 0.85), single-linkage algorithm for hierarchical clustering (–clusterAlg = single), and filtered nucmer alignments for secondary clustering comparisons (–S_algorithm = ANImf). Representative sequences were selected based exclusively on length (−N50W = 0, sizeW = 1). Competitive read recruitment against the dereplicated database of vOTUs was performed with Bowtie 2 v2.4.2 (79) in sensitive mode, and the resulting alignments were sorted and indexed with SAMtools v1.11 (80). We used CoverM v0.5.0 (https://github.com/wwood/CoverM) to generate two vOTU coverage tables: one displaying the trimmed mean coverage (−m = trimmed_mean) and the other displaying the absolute number of mapped reads (−m = count). In both cases, all vOTUs with <75% horizontal coverage were discarded (–min-covered-fraction = 0.75). We filtered out 773 vOTUs that were exclusively detected in single viromes and removed one virome due to poor vOTU recovery (136 vOTUs compared to a median of 1,562 vOTUs). The final dataset consisted of 43 viromes and 5,315 vOTUs.

#### Microdiversity profiling.

Intrapopulation genetic diversity was characterized with inStrain v1.4.0 (56). First, the Bowtie 2 alignments described above were parsed with the profile module to identify divergent sites within the set of mapped reads assigned to each vOTU. Variants were only called if a site had a minimum coverage of five reads. We then used the compare module to calculate average nucleotide identities between sample-specific consensus sequences, which were reconstructed based on the most common allele detected at each variant site. Pairwise comparisons were considered for downstream analyses only if more than 25% of the vOTU sequence length was covered by the profile module in both samples (percent_genome_compared > 0.25).

#### Gene-sharing network construction.

We used Prodigal v2.6.3 (81) in metagenome mode to predict protein content for each dereplicated vOTU and used the resulting amino acid file to construct a gene-sharing network with vConTACT2 v0.9.19 (58). The protein alignment step was performed with Diamond (82), and the protein cluster step was calculated with the MCL algorithm (83). The NCBI RefSeq database of bacterial and archaeal viral genomes (v85) was included as a reference. Layouts used to visualize the resulting network were calculated with the Fruchterman–Reingold algorithm implemented in the GGally package (84).

#### Detection and classification of 16S rRNA gene fragments in virome libraries.

As previously described (14), we used SortMeRNA v4.2.0 (85) against representative versions of the bacterial and archaeal SILVA database v132 (86) to recover reads containing 16S rRNA gene sequences from the set of quality-filtered virome reads. We assigned taxonomy with the RDP classifier (87) using the RDP database v18 (88) as reference. A count table was generated from the resulting hierarchical file with the hier2phyloseq() function from the RDPutils package (89).

#### Processing of 16S rRNA gene amplicon libraries.

Assembly of paired-end reads into single sequences was performed with PANDAseq v2.9 (90), followed by chimeric sequence removal with usearch v6.1 (91). OTU clustering was performed at a 97% sequence identity threshold with the QIIME (92) implementation of UCLUST v1.2.22 (91) following the open reference protocol against the SILVA database v132 (86). For consistency with 16S rRNA gene analysis performed on viromes, representative sequences were reannotated with the RDP classifier (87) using the RDP database v18 (88) as reference. After discarding singletons and OTUs also detected in the blank library, the final dataset consisted of 53,854 OTUs.

### Data Analysis.

All statistical analyses were conducted using R v3.6.3 (93). Unless otherwise noted, all viral analyses were performed on the trimmed mean coverage vOTU table. For vOTU and 16S OTU profiles, Bray–Curtis dissimilarities were calculated on log-transformed relative abundances with the vegdist() function from vegan v2.5-7 (94). PERMANOVAs were performed with the adonis() function from vegan v2.5-7 (94).To calculate the environmental distance, we first computed the z-score for each soil chemistry variable and then used the dist() function to determine the Euclidean distances between pairs of samples. Principal coordinates analyses were performed with the pcoa() function from ape v5.4-1 (95). Pearson’s correlation tests evaluating the association of spatial distance with Bray–Curtis similarity, community overlap, environmental distance, edaphic variables, and vOTU microdiversity were performed using the cor.test() function with the alternative parameter set to “two.tailed.” The associated linear regression slope was calculated with the lm() function. In all cases, spatial distance between pairs of samples was measured as the length of the line connecting the centers of the corresponding plots. To remove any effect of time point on our spatial correlation analyses, we excluded all pairwise comparisons between samples collected at different time points. For correlation analyses involving multiple comparisons (edaphic variables and microdiversity), *P* values were corrected with the Holm algorithm. Indicator species analysis was performed with the multipatt() function from indicspecies v1.7.9 (96). For this analysis, we divided the dataset into two groups, one with the T2-50 viromes and the other with the rest of the samples, and we identified vOTUs significantly associated with each group. We used the lm() function to fit linear models evaluating the effect of collection time point and watering treatment on beta-diversity (as captured by individual principal coordinates) and gravimetric soil moisture. We then used the glht() function from the multcomp package (97) to perform Tukey’s range tests. We used the pairwise.wilcox.test() function to perform pairwise Wilcoxon rank-sum tests to assess the effect of collection time point and watering treatment on the relative abundances of actinobacteria 16S rRNA gene profiles from total DNA and virome DNA. To determine the relative enrichment of vOTUs along the horizontal field transect, we performed a differential abundance analysis with DESeq2 (98), using vOTU nonnormalized count tables as input. In particular, we used the DESeq() function to implement negative binomial generalized models to test the effect of the position of each plot on the abundance of individual vOTUs and used the effect size to rank each viral population. All plots were generated with ggplot2 (99).

#### Local neighborhood enrichment.

To assess whether vOTUs detected as indicator species of T2-50 samples tended to share similar genomic attributes, we adapted a previously described algorithm designed to systematically assess the distribution of traits in biological networks (59). This algorithm consists of two main steps: 1) For each node in the network, determine a local neighborhood comprised of all nodes that can be directly or indirectly reached via an edge path with a length shorter than a defined threshold, and 2) for each local neighborhood, assess the overrepresentation of a particular attribute among its members. In this study, we used the gene-sharing network generated by vConTACT2 (58), in which nodes represent vOTUs, edges indicate a significant overlap in the predicted content between vOTUs, and edge scores denote the statistical significance of the associated overlap (expressed as −log10 *P* value). To determine the distance threshold for local neighborhoods, we first calculated the length of the weighted shortest path for each possible pair of nodes in the network and then identified the first percentile. We performed this step with the distances() function from the igraph package (100), using the reciprocal of the edge scores assigned by vConTACT2 as edge weights. We explored the distribution of the following node attributes across the network: 1) enrichment or 2) depletion in T2-50 samples. To assess the overrepresentation of each of these traits in each of the local neighborhoods, we performed hypergeometric tests using the phyper() function with the “lower.tail” parameter set to false. Local neighborhoods with less than 10 nodes were not considered for the overrepresentation analyses. Multiple comparisons correction was performed with the Holm algorithm.

## Supplementary Material

Supplementary File

Supplementary File

## Data Availability

All raw sequences have been deposited in the NCBI Sequence Read Archive under the BioProject accession PRJNA818793 (101). The database of dereplicated vOTUs is available at https://zenodo.org/record/7076890#.Y1BMPEzMJPY (102). All scripts and intermediate files are available at https://github.com/cmsantosm/HoplandViromes (103).
