# Supplementary material for: Spatial turnover of soil viral populations and genotypes overlain by cohesive responses to moisture in grasslands
Source: Proc Natl Acad Sci U S A. 2022 Nov 2;119(45):e2209132119. doi: 10.1073/pnas.2209132119 (PMC9659419; doi:10.1073/pnas.2209132119)
Supplement: Supplementary File [file pnas.2209132119.sapp.pdf]

PNAS

Supporting Information for

Spatial turnover of soil viral populations and genotypes overlain by cohesive responses to moisture in grasslands

Christian Santos-Medellín, Katerina Estera-Molina, Mengting Yuan, Jennifer Pett-Ridge, Mary K. Firestone, Joanne B. Emerson

Corresponding author: Joanne B. Emerson
Email: jbemerson@ucdavis.edu

This PDF file includes:

Figures S1 to S10
Tables S1 to S3
Legend for Dataset S1

Other supporting materials for this manuscript include the following:

Dataset S1

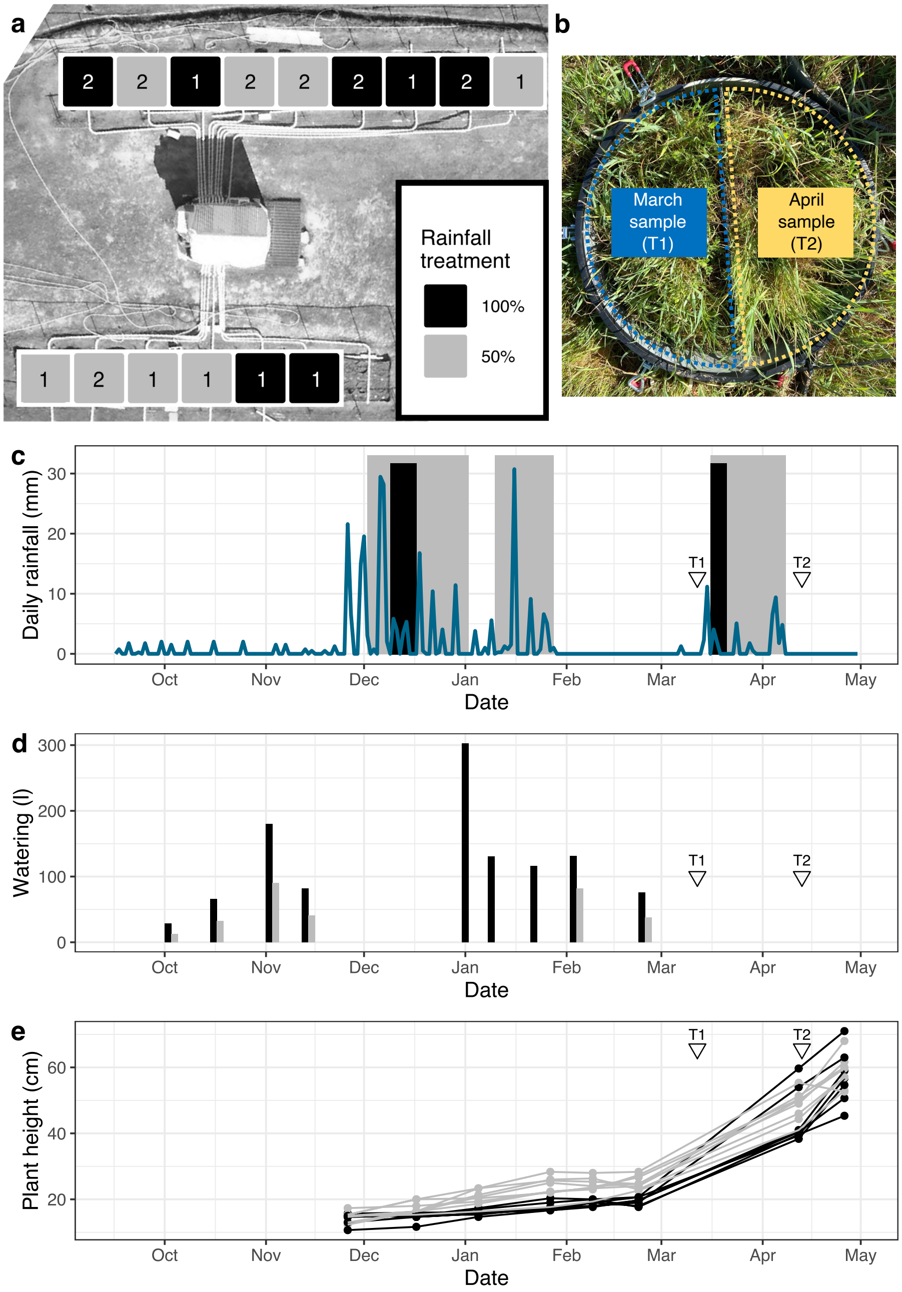

Supplementary Figure 1. (a) Aerial view of the field site. Squares mark the locations of individual plots, and the numbers indicate the number of circular subplots within each plot from which samples were collected. Color indicates the rainfall manipulation treatment for each plot. **(b)** Representative example of a circular subplot. Plexiglass dividers segmented subplots into two halves; soil samples were collected by destructively harvesting one half at each time point. **(c)** Daily precipitation (blue line) at the field site preceding, during, and shortly after the 2020 sample collection time points (T1 and T2). Background blocks (colored by treatment, as in panel **(a)**) indicate periods when the rainfall-excluding shelters were deployed for each treatment. **(d)** Differential watering events during the months preceding sample collection. Each bar indicates the amount of water added to individual plots through irrigation, based on their assigned watering regime (colored as in panel **(a)**). **(e)** Growth patterns of *Avena barbata* during the 2020 growing season. Each line displays the average height of *A. barbata* in a single plot, colored according to precipitation treatment, as in panel **(a)**. In panels **(c)** - **(e)**, inverted triangles mark the two collection time points.

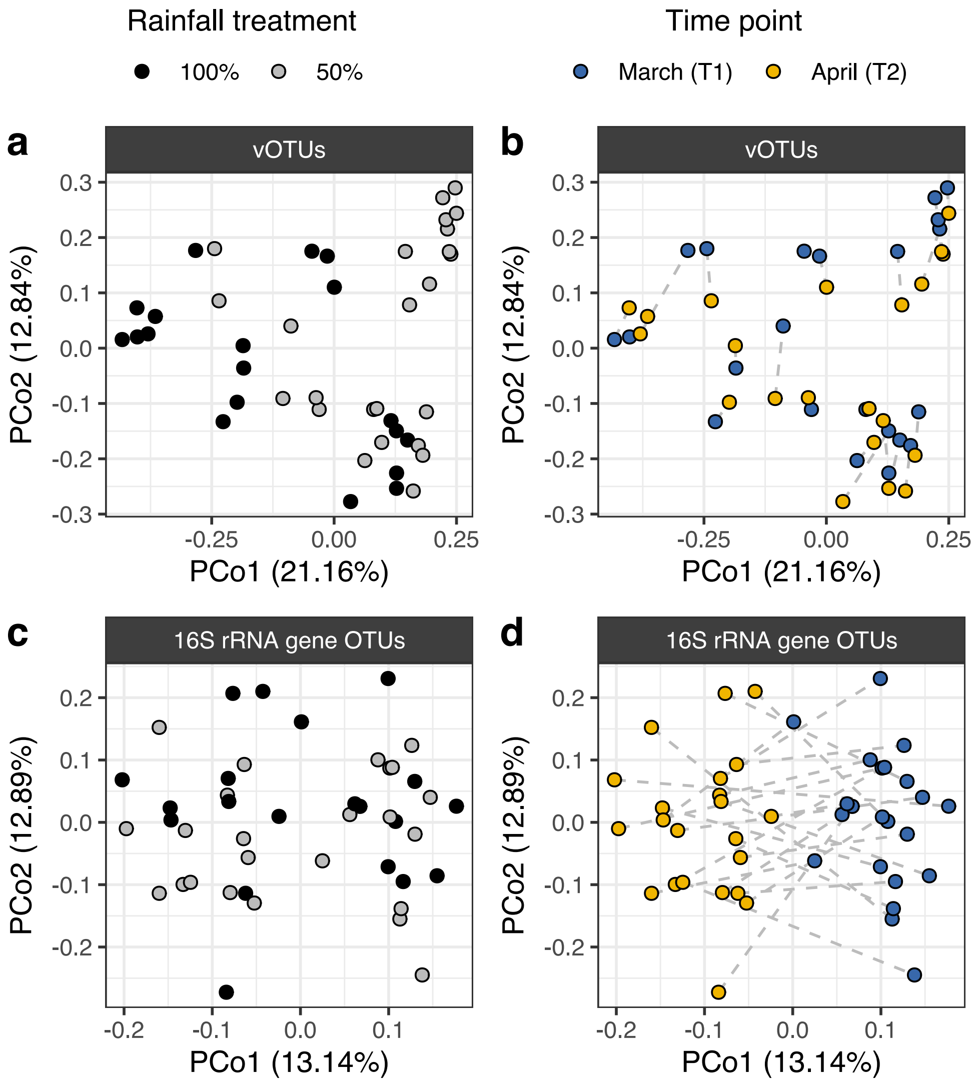

Supplementary Figure 2. First and second axes of unconstrained analyses of principal coordinates performed on **(a,b)** vOTU and **(c,d)** 16S rRNA gene OTU Bray-Curtis dissimilarities. Color in panels **(a,c)** represents the rainfall treatment while color in panels **(b,d)** represents collection time point. In panels **(b,d)**, dotted lines connect samples collected from the same subplot at different time points. In all plots, axis labels indicate the percentage of total variance explained.

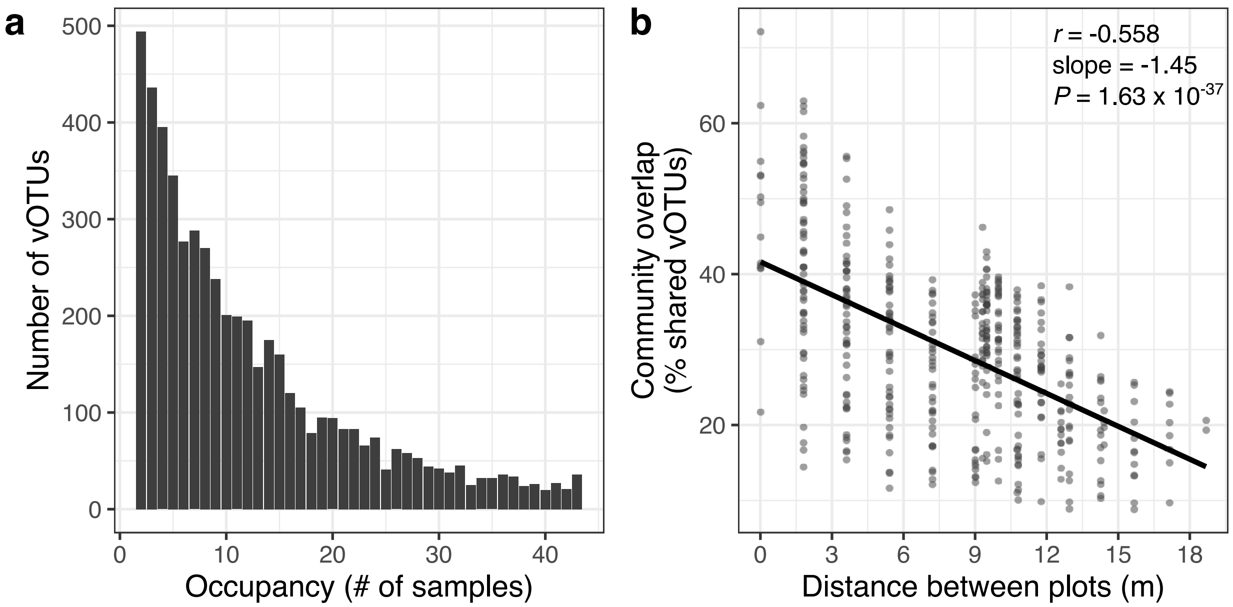

Supplementary Figure 3. (a) Number of vOTUs detected at each occupancy level. **(b)** Relationship between the percentage of vOTUs shared across pairs of samples and spatial distance between plots. Each point represents a pair of samples and the spatial distance between them was measured as the length of the line connecting the centers of the corresponding plots. The trend line displays the least squares linear regression model. Inset statistics correspond to the Pearson's correlation coefficient (*r*), the linear regression slope, and the associated *P*-value.

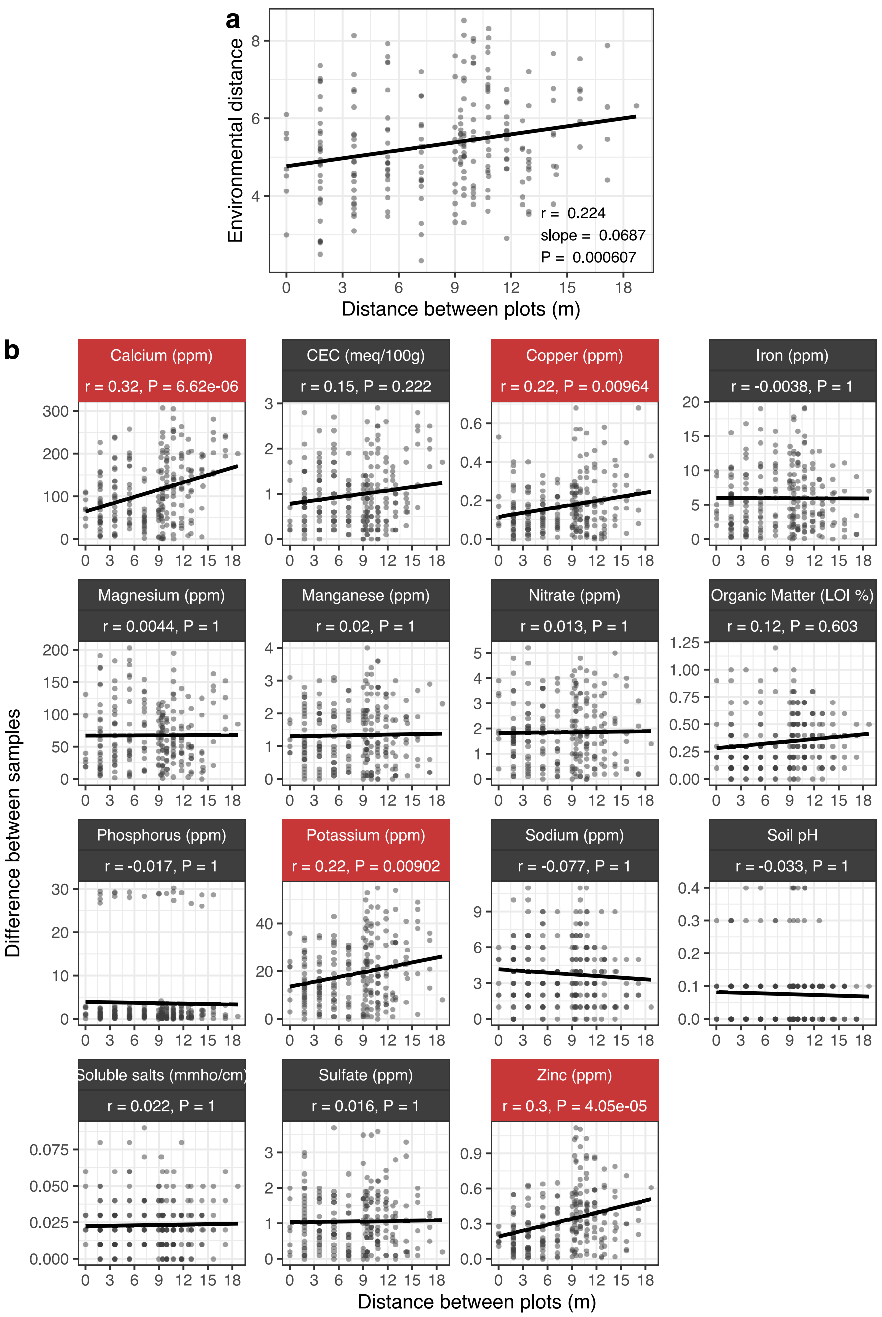

Supplementary Figure 4. (a) Relationship between environmental distance and spatial distance across March (T1) samples, the data subset for which soil abiotic properties were measured. The environmental distance was computed by z-transforming 15 edaphic variables and then calculating their pairwise Euclidean distances. **(b)**. Spatial trends displayed by each of the individual variables used to compute the environmental distance displayed in **(a)**. Each facet corresponds to a single variable with the y-axis indicating the absolute values of the differences between pairs of samples. In both **(a)** and **(b)**, each point represents a pair of samples and the spatial distance between them was measured as the length of the line connecting the centers of the corresponding plots. Trend lines display the least squares linear regression models. Statistics correspond to Pearson's correlation coefficient (*r*) and associated *P*-value. Variables with a significant correlation in **(b)** are highlighted in red. Abbreviations displayed in the facet names correspond to ppm = parts per million; meq/100g = milliequivalents per 100 grams of soil; LOI% = percent weight loss on ignition; mmho/cm = millimhos per centimeter.

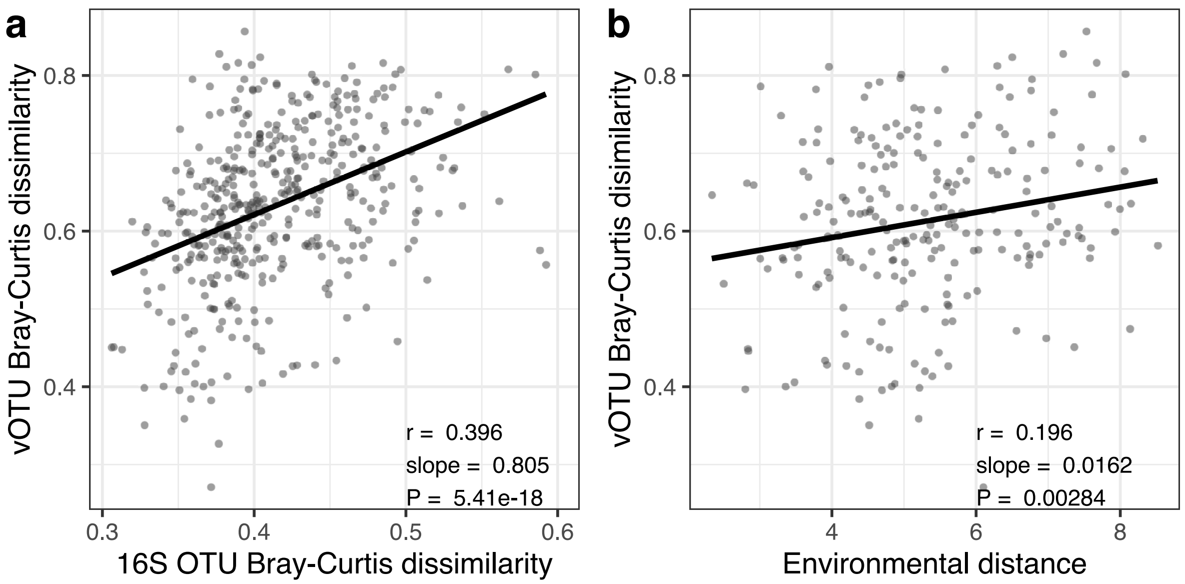

Supplementary Figure 5. (a) Relationship between vOTU Bray-Curtis dissimilarity and 16S rRNA gene OTU Bray-Curtis dissimilarity. **(b)** Relationship between vOTU Bray-Curtis dissimilarity and environmental distance. The environmental distance was computed by z-transforming 15 edaphic variables and then calculating their pairwise Euclidean distances. In both panels, each point represents a pair of samples. In panel **(a),** pairs of samples involving different time points were excluded from these analyses. In panel **(b)**, only pairs of samples involving March samples were used as this was the only time point for which soil abiotic properties were measured. In both panels, trend lines display the least squares linear regression models. Statistics correspond to Pearson's correlation coefficient (*r*) and associated *P*-value.

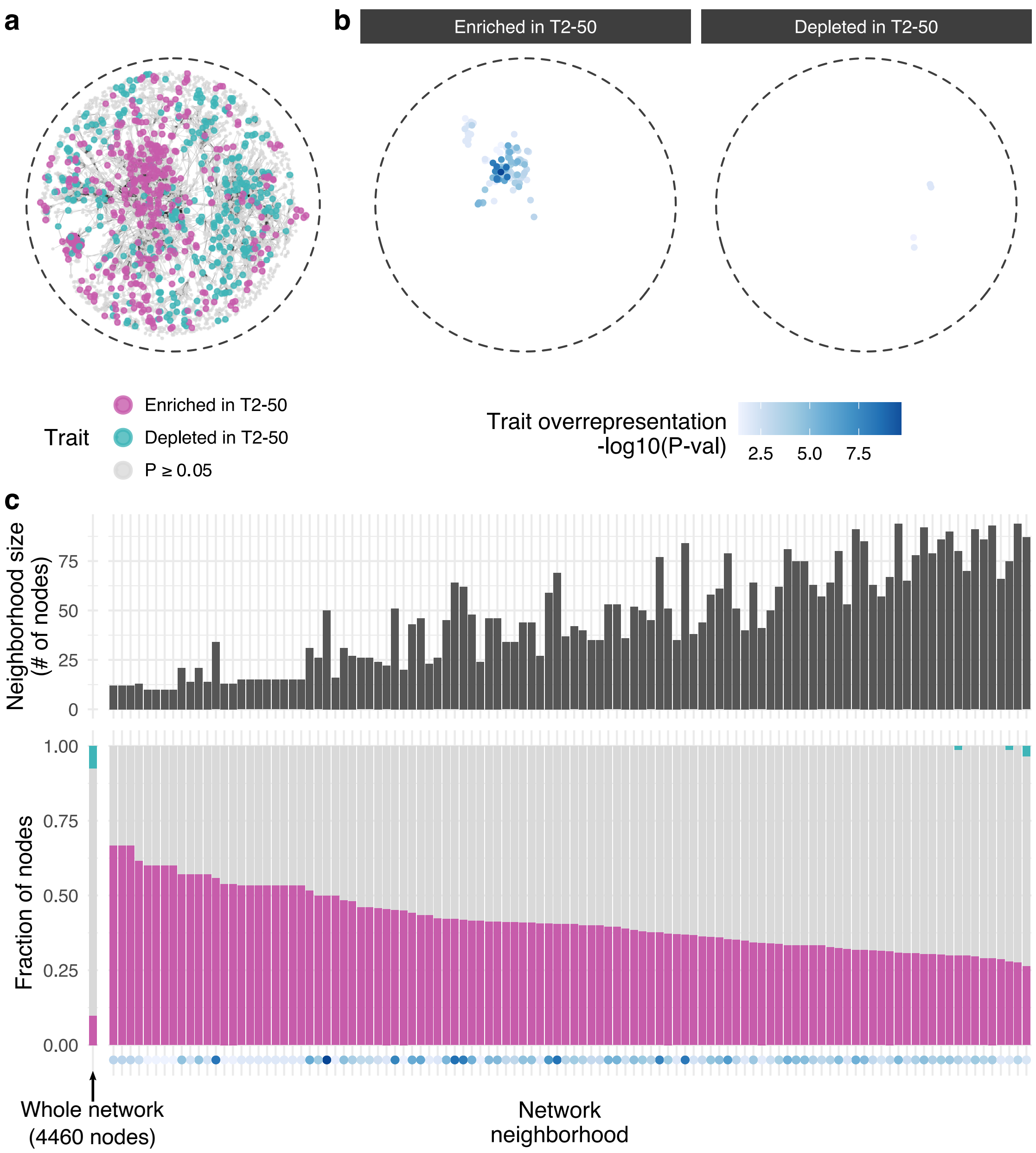

Supplementary Figure 6. (a) Gene-sharing network showing significant overlaps in predicted protein content (edges) between vOTUs (nodes). Node color shows the trait assignment for each vOTU, i.e., whether the vOTU was an indicator species enriched or depleted in T2-50 samples or not an indicator species. **(b)** Distribution of local neighborhoods with a significant overrepresentation of vOTUs enriched (left facet) or depleted (right facet) in T2-50 samples across the network. Each colored point denotes the center of a significant local neighborhood, representing a total of 10 to 94 vOTUs per point. The color gradient indicates the extent of the significance of trait overrepresentation in that neighborhood, with all shades of blue indicating significance and darker shades showing greater significance. Network visualization layout was generated with the Fruchterman-Reingold algorithm. **(c)** Size (upper panel) and trait composition (lower panel) of local neighborhoods with a significant overrepresentation of vOTUs enriched in T2-50 viromes. In the lower panel, each stacked bar plot shows the fraction of indicator vOTUs within a single neighborhood, with the leftmost bar corresponding to the entire network. Dots at the bottom display the statistical significance of the overrepresentation of T2-50-enriched vOTUs in each local neighborhood, following the same color scheme as panel **(b)**.

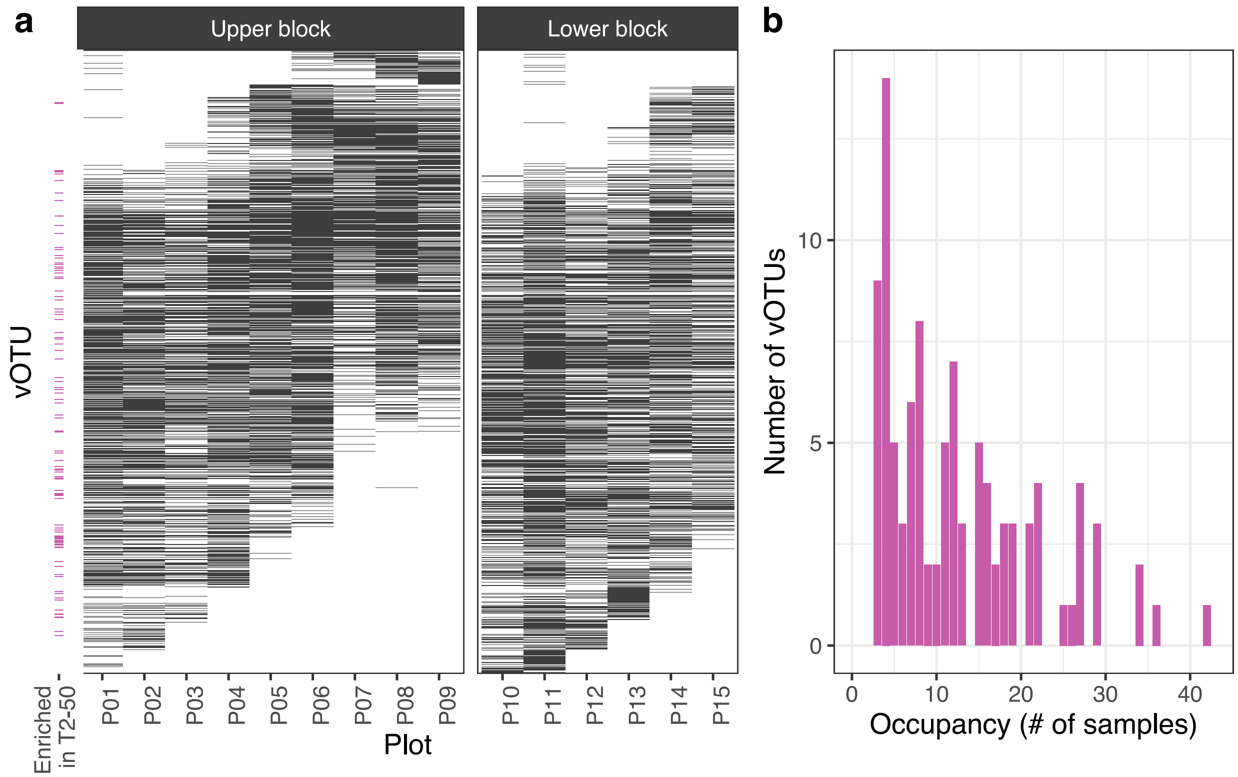

Supplementary Figure 7. (**a)** Distribution of all 5,315 vOTUs detected in this study across the 15 field plots. Each row represents a single vOTU, and its position along the y-axis is determined by its relative enrichment along the field: vOTUs towards the bottom of the y-axis tended to be more enriched on the North-West (left side of the field in **Figure 1a**), while vOTUs towards the top tended to be more enriched on the South-East (right side of the field). The pink tick marks on the left side highlight the indicator vOTUs that were enriched in T2-50 samples and that were part of the subnetwork identified in Figure 3e. **(b)** The occupancy spectrum of the indicator vOTUs highlighted in panel **(a)**.

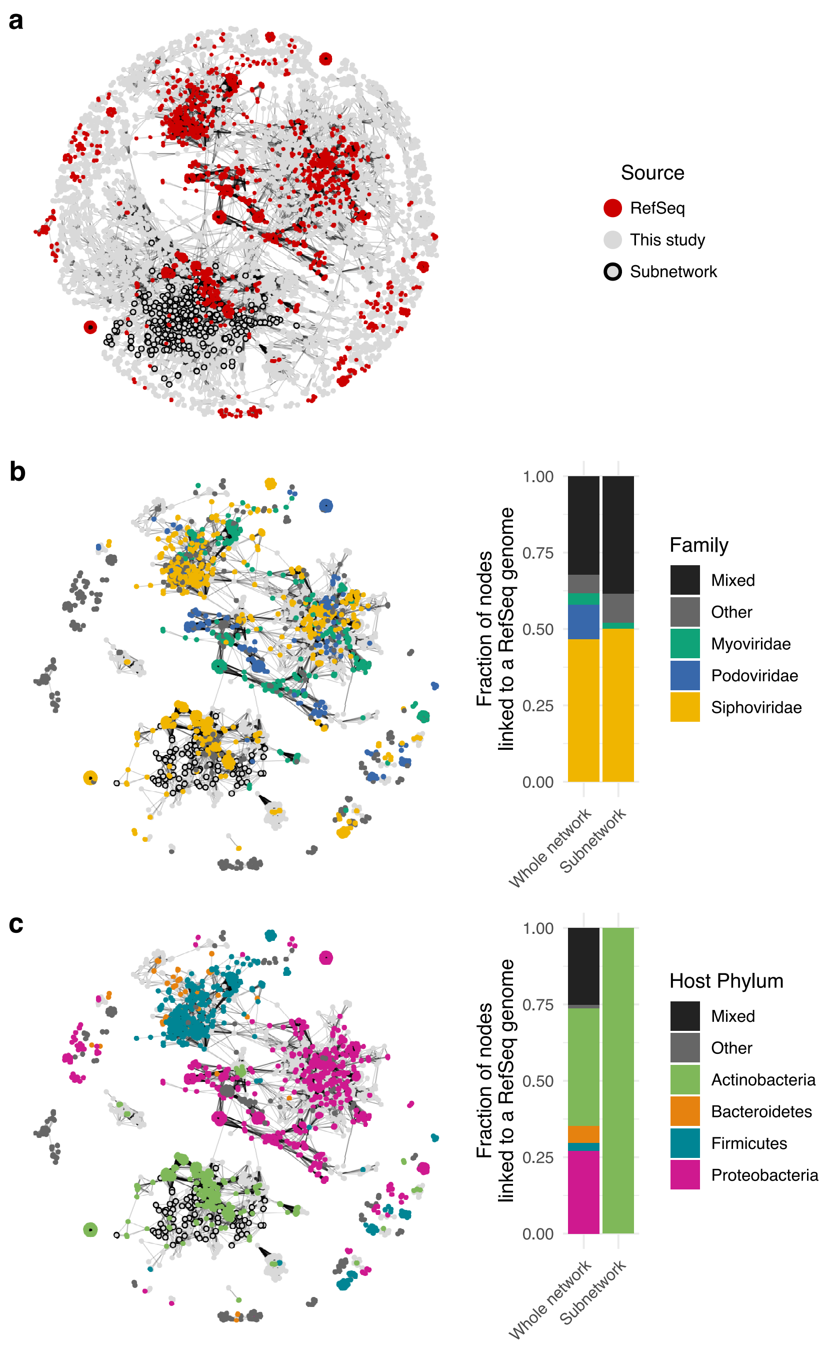

Supplementary Figure 8. (a) Gene-sharing network of vOTUs detected in our study (gray nodes) and prokaryotic virus genomes in RefSeq (red nodes). Edges indicate a significant overlap in the predicted protein content between two viral sequences. The subnetwork highlighted with outlined nodes shows all vOTUs that were part of a local neighborhood with a significant overrepresentation of vOTUs enriched in T2-50 samples (the same nodes outlined in Figure 3d). **(b-c)** The same network, but only showing RefSeq genomes and the subset of vOTUs from our study (light grey) linked by at least one edge to at least one RefSeq genome. Color indicates **(b)** the virus family or **(c)** the host phylum for each RefSeq genome. Accompanying bar plots show the proportion of vOTUs with at least one significant link to a RefSeq genome, with separate bars for the full network and the outlined subnetwork. If a vOTU was linked to multiple RefSeq genomes with differing viral **(b)** or host **(c)** taxonomic classifications, it was labeled as "Mixed". Network visualization layout was generated with the Fruchterman-Reingold algorithm.

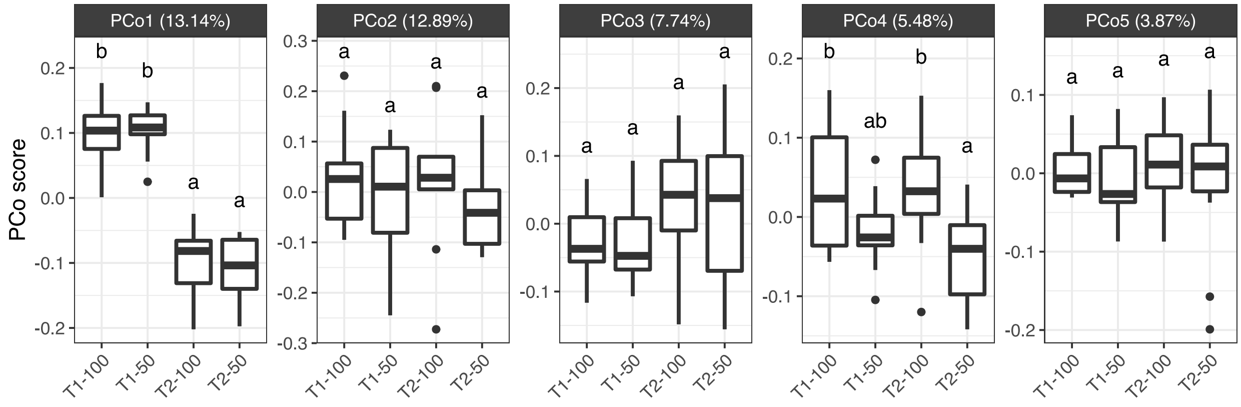

Supplementary Figure 9. Distribution of scores along the first five axes of a principal coordinates analysis performed on Bray-Curtis dissimilarities from 16S rRNA gene OTU profiles. Samples are organized by collection time point (T1 and T2) and precipitation treatment regime (100% and 50%). Boxes display the median and interquartile range (IQR), and data points farther than 1.5x IQR from box hinges are plotted as outliers. Letters indicate significantly different groupings ($P < 0.05$), as determined by Tukey's tests computed for each axis.

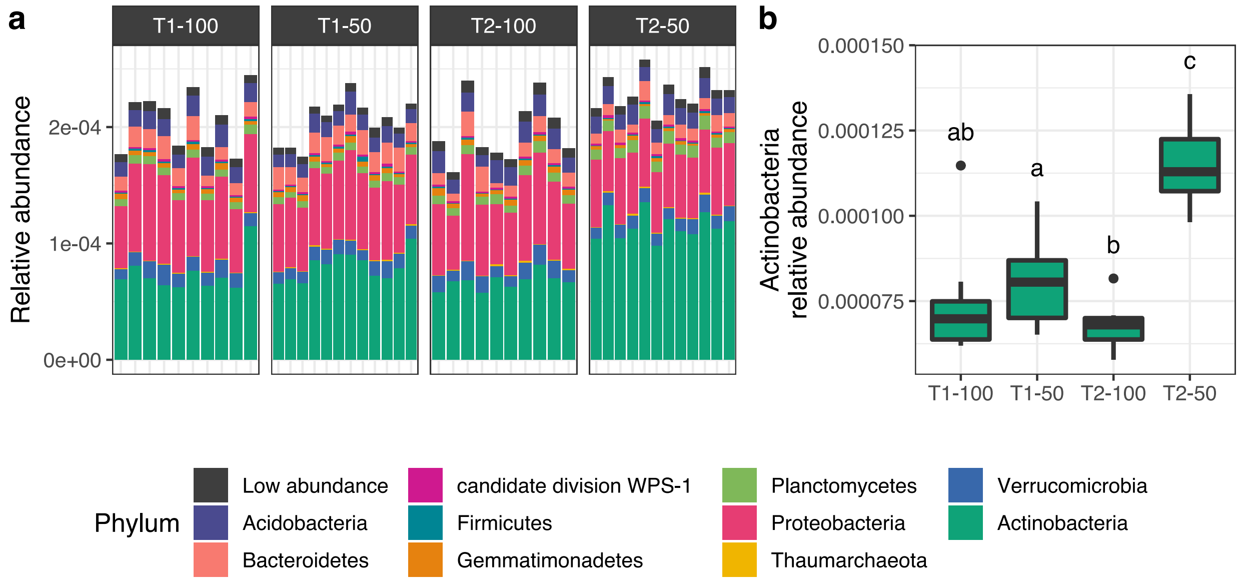

Supplementary Figure 10. Abundance patterns of actinobacteria in relic DNA profiles. (a) Phylum abundances in 16S rRNA gene profiles from virome DNA libraries. Each stacked bar plot corresponds to a sample, and the 10 most abundant phyla are colored. All other phyla are grouped in the 'Low abundance' category. **(b)** Relative abundances of Actinobacteria in virome DNA libraries. Samples are organized by collection time point (T1 and T2) and precipitation treatment regime (100% and 50%). Boxes display the median and interquartile range (IQR), and data points farther than 1.5x IQR from box hinges are plotted as outliers. Letters above boxes indicate significantly different groupings ($P < 0.05$), as determined by pairwise Wilcoxon's rank-sum tests. For both panels, relative abundances were normalized to the total number of reads in each virome profile.

Supplementary Table 1. Permutational multivariate analyses of variance (PERMANOVA) testing the effect of plot position (left-right and top-bottom position based on their location in field - Figure 1a), collection time point, and rainfall treatment on vOTU community composition.

	df	SumsOfSqs	MeanSqs	F.Model	R2	p.value
**PlotPosition_LeftRight**	1	1.678788199	1.678788199	12.21747777	0.189165618	0.001
**PlotPosition_TopBottom**	1	1.000170845	1.000170845	7.278800904	0.11269911	0.001
**Timepoint**	1	0.438827975	0.438827975	3.193595849	0.049447074	0.002
**WaterTreatment**	1	0.535381434	0.535381434	3.896269207	0.060326704	0.001
**Residuals**	38	5.221532036	0.137408738	NA	0.588361494	NA
**Total**	42	8.874700488	NA	NA	1	NA

Supplementary Table 2. Permutational multivariate analyses of variance (PERMANOVA) testing the effect of plot position (left-right and top-bottom position based on their location in field - Figure 1a), collection time point, and rainfall treatment on 16S rRNA gene OTU community composition.

	df	SumsOfSqs	MeanSqs	F.Model	R2	p.value
**PlotPosition_LeftRight**	1	0.258783621	0.258783621	3.175665432	0.061542887	0.001
**PlotPosition_TopBottom**	1	0.163276681	0.163276681	2.003651202	0.038829808	0.008
**Timepoint**	1	0.505390056	0.505390056	6.201898429	0.120189844	0.001
**WaterTreatment**	1	0.099387761	0.099387761	1.21963776	0.023636	0.144
**Residuals**	39	3.178093353	0.081489573	NA	0.755801462	NA
**Total**	43	4.204931472	NA	NA	1	NA

Supplementary Table 3. List of vOTUs displaying a signficant Pearson's correlation between consensus average nucleotide identity (ANI) and spatial distance. The list is arranged by significance (lowest to highest Holm-adjusted P-values). The second column provides the vOTU IDs used for the 5 vOTUs highlighted in Figure 2d.

vOTU_ID	alternative_ID	d.f.	r	p.value	p.adj
**V35_VIR_S449_L004_23157**	vOTU E	350	-0.653137924	3.50E-44	4.41E-42
**V35_VIR_S449_L004_10162**	vOTU B	293	-0.511630397	4.42E-21	5.53E-19
**V34_VIR_S441_L004_86449**	vOTU C	370	-0.441329516	3.66E-19	4.53E-17
**V18_VIR_S478_L004_60094**	vOTU D	116	-0.679422023	2.73E-17	3.36E-15
**V17_VIR_S462_L004_101191**	vOTU A	287	-0.436813529	6.79E-15	8.28E-13
**V38_VIR_S457_L004_35080**	NA	419	-0.359539071	2.72E-14	3.29E-12
**V30_VIR_S468_L004_2448**	NA	368	-0.367830517	2.69E-13	3.22E-11
**V36_VIR_S453_L004_11627**	NA	282	-0.405661399	1.12E-12	1.34E-10
**V21_VIR_S477_L004_23265**	NA	238	-0.426014906	5.32E-12	6.28E-10
**V21_VIR_S477_L004_44604**	NA	280	-0.395141121	5.64E-12	6.60E-10
**V24_VIR_S461_L004_23188**	NA	254	-0.406230583	1.36E-11	1.58E-09
**V43_VIR_S474_L004_483**	NA	306	-0.346748007	3.95E-10	4.55E-08
**V15_VIR_S452_L004_89012**	NA	170	-0.407104687	2.99E-08	3.41E-06
**V02_VIR_S451_L004_67832**	NA	274	-0.323166348	3.96E-08	4.48E-06
**V43_VIR_S474_L004_92062**	NA	337	-0.29022617	5.28E-08	5.92E-06
**V01_VIR_S446_L004_70401**	NA	210	-0.354506978	1.13E-07	1.25E-05
**V35_VIR_S449_L004_80702**	NA	295	-0.29925158	1.47E-07	1.62E-05
**V04_VIR_S455_L004_78422**	NA	408	-0.25585501	1.50E-07	1.64E-05
**V03_VIR_S447_L004_13294**	NA	212	-0.328012863	9.26E-07	0.000100008
**V04_VIR_S455_L004_71169**	NA	340	-0.257129639	1.44E-06	0.000154366
**V39_VIR_S450_L004_21459**	NA	260	-0.262089495	1.73E-05	0.001832137
**V18_VIR_S478_L004_11025**	NA	242	-0.261263328	3.59E-05	0.003774335
**V28_VIR_S460_L004_63708**	NA	296	-0.23158494	5.44E-05	0.005655097
**V16_VIR_S471_L004_41313**	NA	243	-0.254547017	5.57E-05	0.00573269
**V05_VIR_S469_L004_16341**	NA	335	-0.215005475	6.92E-05	0.007060705
**V26_VIR_S440_L004_96825_fragment_2**	NA	346	-0.200364136	0.00016821	0.016989202
**V09_VIR_S458_L004_123193**	NA	363	-0.183313385	0.000431452	0.043145181

Dataset legends

Dataset S1. List of dereplicated vOTUs used in this study.
